# Intraventricular Meningioma: A Case Report of a Common Tumor in an Uncommon Location

**DOI:** 10.7759/cureus.51647

**Published:** 2024-01-04

**Authors:** Shaden S AlMousa

**Affiliations:** 1 Radiology, King Faisal University, AlAhsa, SAU

**Keywords:** dural tail sign, magnetic resonance imaging, lateral ventricle tumour, headache, meningioma

## Abstract

Intraventricular meningiomas are uncommon neoplasms originating within the ventricular system of the brain, constituting a rare subset of central nervous system tumors. Their unique location poses diagnostic challenges, and understanding their clinical manifestations is imperative for effective management. We present the case of a 56-year-old female who sought medical attention due to persistent, severe headaches localized in the occipital region. Laboratory investigations demonstrated normal values for a complete blood count, electrolytes, and liver function tests. Magnetic resonance imaging revealed a well-defined mass in the left occipital horn of the lateral ventricle, indicative of an intraventricular meningioma. Surgical resection of the intraventricular meningioma resulted in the resolution of the patient's headaches. This case contributes valuable insights into the diagnostic challenges posed by intraventricular meningiomas.

## Introduction

Intraventricular meningiomas are rare neoplasms originating within the ventricular system of the brain, accounting for only 1-3% of all meningiomas [1]. These tumors, arising from the arachnoid cells of the meninges, exhibit distinct clinical and radiological characteristics, necessitating a tailored diagnostic and therapeutic approach [1,2]. The present case involves a 56-year-old female who sought medical attention due to persistent, severe headaches localized in the occipital region. Headaches of this nature prompted a comprehensive investigation, leading to the identification of an intraventricular meningioma.

While meningiomas are commonly associated with the dura mater, intraventricular variants pose unique challenges in diagnosis and management. The clinical presentation often mirrors that of more common intracranial pathologies, emphasizing the significance of careful evaluation and a high index of suspicion [1,2]. This case underscores the need for a multidisciplinary approach involving neurologists, neurosurgeons, and neuroradiologists to navigate the complexities associated with intraventricular lesions. Given the rare prevalence of intraventricular meningiomas and the variability in their clinical presentations, this case adds valuable insights to the existing literature, shedding light on the difficulties involved in the diagnosis and successful management of this uncommon neurosurgical entity.

## Case presentation

A 56-year-old female presented to the neurology clinic with a chief complaint of persistent, severe headaches over the past three months. The headaches were described as throbbing in nature, predominantly located in the occipital region, and associated with nausea and occasional vomiting. The patient reported a gradual onset and progressive worsening of the headache intensity. She denied any specific triggering factors, such as changes in posture, exertion, or dietary habits. There were no accompanying neurological deficits, visual disturbances, or other focal symptoms noted during the headache episodes.

The patient's past medical history revealed hypertension, which was well-controlled with antihypertensive medications. There were no significant contributory factors, such as recent head trauma, systemic infections, or exposure to neurotoxic substances. The family history was unremarkable for neurological disorders, including brain tumors. Social history was notable for a non-smoking status and an absence of recreational drug use.

On physical examination, the patient appeared well-nourished and in no apparent distress. Vital signs, including blood pressure, were within normal limits. Neurological examination demonstrated intact cranial nerve function, normal strength, sensation, and coordination in all extremities. Fundoscopic examination did not reveal any papilledema or other abnormalities. The remainder of the systemic examination was unremarkable.

Given the persistent nature of the headaches and the absence of identifiable triggers, further work-up was initiated. Routine laboratory investigations, including a complete blood count, electrolytes, and liver function tests, were all within normal limits (Table 1).

**Table 1:** Initial laboratory investigations

Laboratory Test	Result	Reference Range
Hemoglobin	13.2 g/dL	12.0 - 15.5 g/dL
White Blood Cell Count	8.5 x 10^3^/μL	4.0 - 11.0 x 10^3^/μL
Platelet Count	220 x 10^3^/μL	150 - 450 x 10^3^/μL
Sodium	140 mmol/L	135 - 145 mmol/L
Potassium	4.0 mmol/L	3.5 - 5.0 mmol/L
Blood Urea Nitrogen	12 mg/dL	7 - 20 mg/dL
Creatinine	0.8 mg/dL	0.6 - 1.2 mg/dL
Alanine Aminotransferase	25 U/L	7 - 56 U/L
Aspartate Aminotransferase	30 U/L	5 - 40 U/L
Alkaline Phosphatase	70 U/L	35 - 104 U/L
Total Bilirubin	0.8 mg/dL	0.2 - 1.2 mg/dL

**Figure 1:**
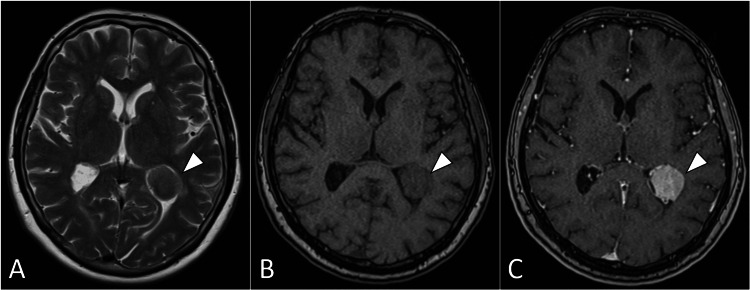
Axial brain MRI images featuring T2-weighted image (A), T1-weighted image (B), and post-contrast image (C). The images reveal a distinct intraventricular lesion (arrowhead) situated in the occipital horn of the left lateral ventricle, exhibiting pronounced solid contrast enhancement—a characteristic indicative of meningioma. MRI: magnetic resonance imaging

Magnetic resonance imaging of the brain was subsequently performed, revealing a well-defined mass within the occipital horn of the left lateral ventricle. It has a similar signal intensity to that of grey matter and demonstrates solid homogeneous enhancement, suggestive of an intraventricular meningioma (Figure 1).

The differential diagnosis considered at this stage included other intraventricular lesions such as ependymoma, choroid plexus papilloma, and central neurocytoma. However, the characteristic radiological features supported the diagnosis of intraventricular meningioma.

**Figure 2:**
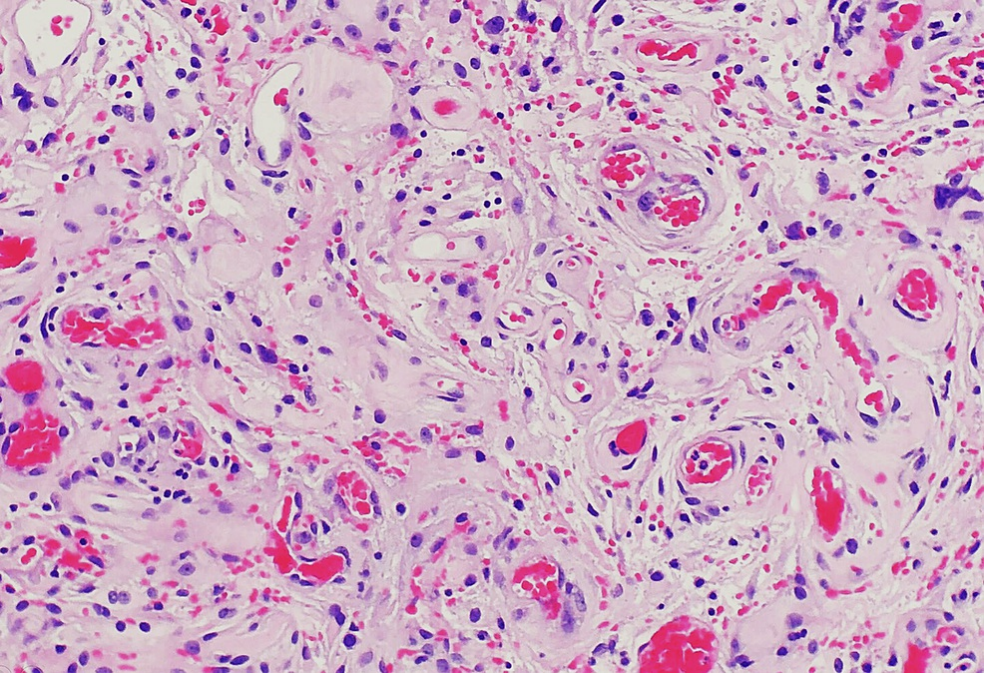
Histopathological analysis of the specimen unveils the distinctive whorled architecture of tumor cells featuring oval nuclei characterized by finely dispersed chromatin, indicative of the characteristic morphology associated with meningeal cells.

The patient was referred to a neurosurgeon for further management. Given the location and size of the meningioma, surgical resection was deemed the most appropriate intervention. The surgical procedure was performed successfully, and the histopathological examination of the excised tissue confirmed the diagnosis of a World Health Organization (WHO) Grade I meningioma (Figure 2). Postoperatively, the patient experienced a gradual resolution of her headaches. Throughout the hospital course, there were no significant complications, and the patient's neurological status remained stable. In subsequent outpatient follow-up visits, the patient continued to show improvement in her symptoms. Regular surveillance will be maintained to monitor for any potential recurrence or complications associated with the meningioma.

## Discussion

Intraventricular meningiomas, although accounting for a small fraction of all meningiomas, present unique challenges due to their location within the cerebral ventricles. These tumors originate from the arachnoid cap cells and can be found along the ventricular system, with a predilection for specific sites such as the lateral and third ventricles [1,2]. Despite their infrequency, the management of intraventricular meningiomas poses significant clinical dilemmas, necessitating a thorough understanding of their characteristics [3].

The clinical presentation of intraventricular meningiomas varies depending on their location and size. Common manifestations include headaches, visual disturbances, hydrocephalus, and focal neurological deficits. Due to the subtle and nonspecific symptoms, early diagnosis is often challenging, leading to delayed intervention and potential complications [2-4].

Accurate diagnosis of intraventricular meningiomas is imperative for effective management. Neuroimaging techniques, including magnetic resonance imaging and computed tomography scans, are pivotal in localizing and characterizing these tumors [1-3]. Our patient's magnetic resonance imaging revealed a well-defined, contrast-enhancing mass within the left occipital horn of the lateral ventricle, a characteristic finding in such cases. Notably, our case lacked the commonly reported "dural tail sign" on magnetic resonance imaging, a finding often associated with meningiomas. The absence of this radiological feature highlights the variability in imaging presentations. While advanced modalities such as magnetic resonance spectroscopy can initially employed to confirm the neoplastic nature, the histopathological examination post-surgical resection ultimately confirmed the diagnosis of a World Health Organization (WHO) Grade I meningioma.

The successful surgical resection of the intraventricular meningioma, as demonstrated in our case, aligns with existing literature advocating for a surgical approach as the primary mode of management for these tumors [3-5]. The absence of significant complications during the hospital course and the patient's subsequent improvement in symptoms further underscore the efficacy of surgical intervention in achieving favorable outcomes. Factors such as tumor size, histological subtype, and extent of resection influence prognosis [3,4].

## Conclusions

In conclusion, the presented case of intraventricular meningioma underscores the diagnostic challenges and therapeutic considerations associated with this rare and intricate neurosurgical entity. Through a multidisciplinary approach encompassing neuroimaging, histopathological analysis, and surgical intervention, we successfully navigated the complexities of the case, demonstrating the importance of a tailored and comprehensive management strategy. Our experience highlights the need for heightened clinical awareness and collaborative efforts among neurosurgeons, radiologists, and pathologists to enhance early detection and optimal treatment outcomes for patients with intraventricular meningiomas.

## References

[1] Nanda A, Bir SC, Maiti T, Konar S (2016). Intraventricular meningioma: technical nuances in surgical management. World Neurosurg.

[2] Ammendola S, Simbolo M, Ciaparrone C (2022). Intraventricular meningiomas: clinical-pathological and genetic features of a monocentric series. Curr Oncol.

[3] Nakamura M, Roser F, Bundschuh O, Vorkapic P, Samii M (2003). Intraventricular meningiomas: a review of 16 cases with reference to the literature. Surg Neurol.

[4] Bertalanffy A, Roessler K, Koperek O, Gelpi E, Prayer D, Neuner M, Knosp E (2006). Intraventricular meningiomas: a report of 16 cases. Neurosurg Rev.

[5] Bhatoe HS, Singh P, Dutta V (2006). Intraventricular meningiomas: a clinicopathological study and review. Neurosurg Focus.

